# Assessment of Occupational Injury among Industrial Workers in the Bahir Dar City of Northwest Ethiopia: Institution-Based Cross-Sectional Study

**DOI:** 10.1155/2021/2793053

**Published:** 2021-03-20

**Authors:** Berhanemeskel Hunegnaw, Mesafint Molla, Yihun Mulugeta, Maru Meseret

**Affiliations:** ^1^School of Public Health College of Medicine and Health Sciences, Bahir Dar University, Bahir Dar, Ethiopia; ^2^Department of Health Informatics, College of Health Sciences, Debre Markos University, Debre Markos, Ethiopia

## Abstract

**Background:**

Ethiopia is one of the economically fastest growing countries in the world. Industries in Ethiopia are booming, and Bahir Dar is one of the industrial zones in the country. The city administration is planning to recruit the majority of the workforce in these industries. However, injuries related to occupations in the industries are not that much studied yet. Therefore, this study aimed to assess the prevalence of occupational injury and its associated factors among industrial workers in the Bahir Dar city of Northwest Ethiopia.

**Methods and Materials:**

Institution-based cross-sectional study design was used. Multistage stratified random sampling technique was employed to select 846 study participants from each stratum (small-, medium-, and large-scale industries). Data were analyzed using Statistical Package for Social Sciences (SPSS), version 21. Binary logistic regression was employed to determine the existence of an association between dependent and independent variables.

**Result:**

A total of 803 participants were included in the study with a response rate of 95%. The mean age of respondents was 28.9 years (SD ± 8 years). Five hundred nine (63.4%) were occupationally injured in the last 12 months. Sex (AOR = 3.66, 95% CI = (2.53–5.29)), employment status (AOR = 7.33, 95% CI = (3.31–16.22)), regular health and safety supervision (AOR = 2.66, 95% CI= (1.79–3.96)), training prior to entry to actual work (AOR = 3.18, 95% CI= (2.14–4.74)), and use of personal protective equipment (PPE) (AOR = 2.48, 95% CI= (1.74–3.56)) were significantly associated with occupational injury.

**Conclusion:**

The prevalence of occupational injury in this research is found to be very high. It is advisable to provide sustainable training and regular occupational health and safety supervision for industrial workers in the city. Moreover, research has to be conducted to know the reasons for the difference in the prevalence of occupational injury between large-, medium-, and small-scale industries.

## 1. Background

Occupational injury is any unintentional physical damage or harm to the body tissue from occupational exposure. This includes abnormal condition or disorder that caused an illness that result in death, loss of consciousness, or work absences by the work environment sustained on the worker in connection with the performance of his/her job but does not include work-related diseases that need exposure assessment or laboratory test examination [1].

It is obvious that occupational injuries are a source of huge human and economic cost, and 4% of global gross domestic product (GDP) was estimated to be lost economically. The burden is not only for the individual worker but also for the organization, community, and societal and national levels at large. Occupational injuries have been known for causing various personal sufferings and diminishing the moral of workers [2–4].

The impact of occupational injury in the greatest concentration of the global workforce is 10 to 20 times higher in developing counties [5]. Moreover, the majority of the global workforce does not have access to occupational health services. Only 5 to 10 percent of the workforce in developing countries and 20 to 50 percent of the workforce in developed countries have access to some kind of occupational health services [6].

The International Labor Organization (ILO) 2018 report indicated that the global number of work-related fatal and nonfatal accidents and diseases does not seem to have changed significantly during the past 10 years. This is mainly due to the globalization process and by the rapid growth of industrialization in relatively poor countries that are not capable of maintaining effective health and safety systems [7].

According to a study conducted among 268 smaller industrial workers in Norway, the injury rate of 317 per 1000 exposed workers was observed in a one-year period [8], while a study conducted in the United States (US) showed that among large industrial workers, 75 per 1000 exposed workers injured per year [9].

In Africa, reports showed that industries are responsible for many types of injuries such as burn injuries on different parts of the body, fracture, and falling and responsible for hospitalization, high cost for treatment, and loss of productivity due to absence from workplace [10]. A workplace accident can have catastrophic effects on the quality of life of the worker and also have a devastating effect on family and friends; as a result, it continues to be a prominent public health concern. Similarly, the costs associated with workplace injuries can be substantial for the injured workers, employers, and the nation [11].

Small- and medium-scale industries employed about 80% of the workforce and contribute over 90% of all industries in developing countries. Workers in these industries are at a greater risk of work-related injuries, chronic illness, stress, and disability or death because of low educational and literacy rates, unfamiliarity with work process and exposures, and inadequate training [12].

Studies that are available on industrial injuries in Africa indicated that occupational injuries appeared with greater frequency and severity. For instance, the study conducted in Zimbabwe showed that the injury rate among small-scale industrial workers was 131 per 1000 exposed workers per year [13], while a similar research conducted in Addis Ababa, the injury rate among 4,462 small-scale industrial workers was 80 per 1000 exposed workers per year [14].

Reports from the Department of Environmental Health of Ministry of Health in the country indicated that among 16,610 large-scale industrial workers in Addis Ababa, a prevalence rate of 723 injuries per 1000 exposed workers was observed [15]. Shreds of evidence all over the world indicated that there is a significant difference in the injury rate between small- and large-scale industries [8, 9, 14, 15].

Countries all over the world are losing most of their economy for other major nonquantifiable costs, such as the insurance and health care costs, that affected individuals face; the indirect costs that companies incur (e.g., the cost of training inexperienced replacement workers, administrative expenses, production bottlenecks, low employee morale); the impact on families and communities; and the inefficiency of having a large proportion of a potentially active workforce being disabled [16].

According to a report from Ethiopia, millions of daily laborers work in big constructions in an unsafe working environment and without supportive and protective equipment. They do not have protective caps, hand gloves, eye glasses, working clothes, shoes, and so on. They work on high-rise buildings standing on old and inclined wooden scaffolds and ladders, and they even transport heavy construction materials on them [17].

Ethiopia is one of the economically fastest growing countries in the world. Industries in Ethiopia are booming; and Bahir Dar is one of the industry zones in the country, and the city administration is planning to recruit the majority of the workforce in these industries. However, injuries related to occupations in the industries are not that much studied yet.

## 2. Methods

*Study Design*. Institution-based cross-sectional study design was conducted. Data were collected from 01 February 2017 to 30 February 2017 within one month.

*Study Area*. The study was carried out in Bahir Dar city administration, which is the capital city of the Amhara region and located 563 kilometers away from Addis Ababa, which is the capital city of Ethiopia. According to the 2014 Amhara Regional State Bureau of Finance and Economic Development (BoFED), Bahir Dar city administration had a total population of 284,020 (male = 134,818 and female = 149,202). Of the total population, 226,713 were living in an urban area. In the city, there were 121, 64, and 25 small-, medium-, and large-scale industries, respectively, which consisted of 6339 workers. Regarding health service infrastructure, there were 2 and 10 public hospitals and health centers in the city, respectively [18].

*Source Population*. All employees working in the production process of the industries found in the Bahir Dar city during the study period were the source population for the study.

*Study Population*. Workers in the selected industries who were directly engaged in the production process and selected to participate in the study were considered the study population.

*Inclusion and Exclusion Criteria*. All employees who were directly engaged in the production process in the selected industries were included in the study, while employees who were not directly involved in the production process such as administrative staff, because by their occupation they were not exposed to occupational injury, and those who were working for less than one year were excluded from the study.

### 2.1. Sample Size Determination and Sampling Procedure

The sample size was determined by using a single population proportion formula considering the “proportion of occupational injury” as a key variable. It was calculated using 48.9% as the proportion of occupational injury among industrial workers in Ethiopia [19], assuming 5% Type I error, 95% confidence interval, 5% nonresponse rate, and a design effect of 2 yields, a total of 846 study participants.

Multistage stratified random sampling technique was employed. First, each industry (small, medium, and large scale) in the city was given a number. A total of 52 industries were proportionally selected from each stratum (30, 16, and 6 from small-, medium-, and large-scale industries, respectively) by using the lottery method. A list of employees working in the production unit was obtained from their payroll, each employee in each industry was given a number, and workers from each industry (85, 264, and 497 from small-, medium-, and large-scale industries, respectively) were selected proportionally using computer-generated random numbers.

### 2.2. Measurements and Definitions

For the purpose of this study, the occupational injury was defined as any personal injury and sickness resulting from an accident in the course of work for the past year before this study. The only incidence of the event for a given time is recorded as an injury [20]. Job satisfaction was also defined as a state of pleasurable emotional feeling reported by the worker as a result of one's job. It is a subjectively perceived response of study participants to their job [20]. Sleep disturbance problem was defined as sleeping less than 5 hours in a 24-hour period [21], and finally, PPE was defined as specialized clothing or equipment worn by employees for protection against health and safety hazards. Workers were classified as those who used PPE when they responded to use PPE that was necessary to be worn during a particular activity [22].

### 2.3. Data Collection and Quality Assurance

Data were collected by an interviewer-administered, structured questionnaire, which was adopted after review of relevant literature. The questionnaire was prepared first in English and translated to Amharic language and then again back-translated into English. Detail information of sociodemographic, work environment, and behavioral variables of industrial workers in the preceding one year was collected by six trained urban health extension workers and three BSc environmental health experts as supervisors.

A pretest was performed in 5% of the sample size in nonstudy areas to check the clarity and appropriateness of the data collection tool. An appropriate amendment was carried out on the questionnaire after collecting the feedback of the pretest. Training on the objective of the study, method of data collection, and interviewing techniques was given for data collectors and supervisors for 2 days by investigators. During the data collection period, adequate supervision was carried out by supervisors and by investigators themselves. Daily on the spot checking of filled questionnaires for errors or any incompleteness on daily basis during data collection was performed.

### 2.4. Data Analysis

The data were entered, checked, and cleaned using the statistical software Epi Info, version 3.5.1. After this, it was exported to SPSS, version 21, statistical package for analysis. Frequencies, percentage, and mean and standard deviation were used for the descriptive analysis. Binary logistic regression was employed to determine the existence of an association between the dependent variable (occupational injury status) and a wide range of independent variables. Variables with *P* value <0.2 were taken for multivariate analysis to see their adjusted effect on the dependent variable. A multivariate analysis existence of association was declared at *P* < 0.05 with a 95% confidence interval.

## 3. Results

### 3.1. Sociodemographic Characteristics of Respondents

A total of 803 participants with a response rate of 95% were included in this study. The mean age of the study participants was 28.9 (SD ± 8) years, and 544 (67.7%) of them were within the age group of 14–29 years. Five hundred ninety-one (73.6%) of the study participants were male. Regarding religion, educational status, and marital status, 749 (93.3%) were orthodox, 378 (47%) were certificate or diploma holders, and 384 (47.8%) were married. The majority (86.6%) of the respondents were permanently employed, where 536 (66.7%) of them had ≤7 years of experience and half of the participants earned a salary of ≤US$ 91 (Table 1).

### 3.2. Working Environment Characteristics of Respondents

The majority of the respondents 567 (70.6%) had pre-employment health and safety training. On the other hand, 190 (23.7%) of the respondents had regular health and safety supervision. Concerning working hour, 743 (92.5%) were working for less than or equal to 48 hours per week, but the rest of the respondents were working for more than 48 hours per week with 47 ± 4.4 mean working hours.

### 3.3. Behavioral Characteristics of Respondents

Among the total study participants, only 8 (1%) were smoking cigarettes, and all of them were smoking every day. Participants were also asked to explain whether they drank alcohol or not, and 350 (43.6%) were drinking alcohol. Of the total alcohol drinkers, 24 (6.9%), 210 (60%), and 116 (33.1%) were drinking alcohol every day, 1–3 days per week, and occasionally, respectively. Moreover, among the total study participants only 16 (2%) were khat chewers, and of the khat chewers, 9 (56.3%), 6 (37.5%), and 1 (6.3%) used to chew every day, 1–3 days a week, and occasionally, respectively.

Of the total study participants, 85 (10.6%) were having a sleeping disorder. Reported reasons for having sleeping disorder were 39 (45.9%) due to working for more than 8 hours a day, 27 (31.8%) due to working at night, 18 (21.2%) due to working more than one task at a time, and 1 (1.2%) due to excessive heat emitted by the work.

The current study shows 704 (87.7%) of respondents reported that they were satisfied with their current job. Regarding PPE, two-thirds of the study participants, 508 (63.3%), were using while they were working. The type of personal protective equipment used by the workers was described as in Table 2.

### 3.4. Prevalence of Occupational Injury

The prevalence of occupational injury was 324 (69.4%), 143 (56.5%), and 42 (50.6%) among large-, medium-, and small-scale industries, respectively, with the overall prevalence of 509 (63.4%). Concerning the frequency of occupational injury, 334 (65.6%) of them were injured <3 times within 12 months, while 175 (34.4%) were injured ≥3 times within 12 months.

Regarding the occurrence time of the injury, 149 (29.3%), 312 (61.3%), and 48 (9.4%) were injured in the morning, afternoon, and evening, respectively. Respondents were also reported that 133 (26%) of them were hospitalized because of occupational injury. Of which, 87 (65.4%) and 46 (34.6%) were hospitalized for >24 and ≤24 hours, respectively. Moreover, 234 (46%) were absent from their job because of problems related to occupational injury.

### 3.5. Affected Body Parts, Type, and Sources of Occupational Injury

**Figure 1:**
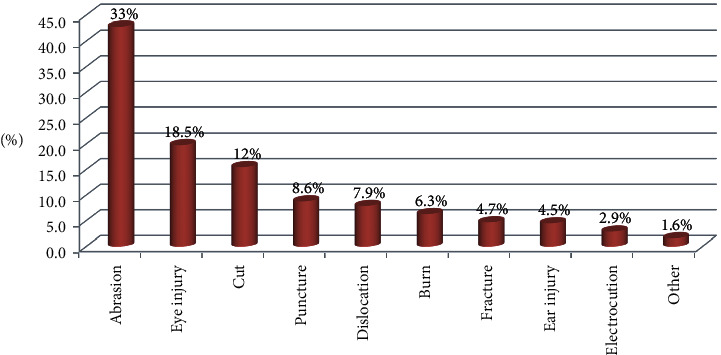
Distribution of types of occupational injury among industrial workers in the Bahir Dar city, 2017 (*n* = 509).

**Figure 2:**
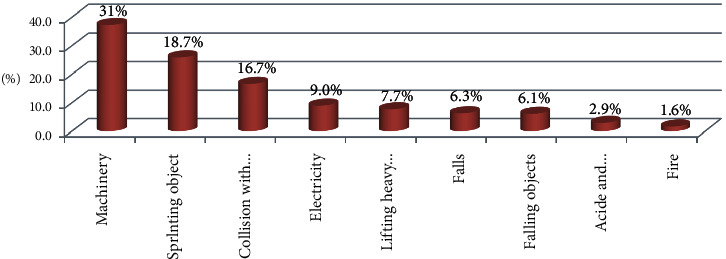
Distribution of sources of occupational injury among industrial workers in the Bahir Dar city, 2017 (*n* = 509).

Among occupationally injured participants, the majority (37.1%) of them had hand injury followed by eye injury 94 (18.5%), but the tooth, knee, and head were with lowest injury accounting 8 (1.6%) for each (Table 3). Regarding the types of injuries, 168 (33%) was abrasion (Figure 1). However, 158 (31%) of the injury was due to machinery followed by 95 (18.7%) of splinting objects (Figure 2).

Respondents were also requested to describe possible reasons for their occupational injury, and the source of injury for the majority (62.9%) of them was the nature of the work, for 226 (44.4%) of them was failed to use PPE, for 24 (4.7%) of them was the condition beyond their capability, for 16 (3.14%) of them was another source, and for 8 (1.6%) of them was new to work and for 8 (1.6%) of them did not remember the source of the injury.

### 3.6. Factors Associated with Occupational Injury

Bivariable analysis was done for each variable to see their crude effect on the dependent variable. Variables such as sex, marital status, type of employment, training, supervision, personal protective equipment use, and experience were found to have *P* value <0.2 with a 95% confidence interval (Table 4). However, at multivariate analysis, only sex, employment status, having training; supervision, and utilization of PPE were associated with occupational injury at *P* value <0.05.

Male workers were 3.66 times more likely to have occupational injury compared with female workers (AOR = 3.66, 95% CI = (2.53–5.29)). Similarly, temporarily employed industry workers were 7.33 times more likely to have occupational injury compared with permanently employed workers (AOR = 7.33, 95% CI = (3.31–16.22)).

This study indicated that those industry workers who were not regularly supervised by health and safety personnel were 2.66 times more likely to have occupational injury compared with their counterparts (AOR = 2.66, 95% CI = (1.79–3.96)). Moreover, workers who did not have pre-employment training regarding occupational health and safety were 3.18 times more likely to have occupational injury compared with their counterparts (AOR = 3.18, 95% CI = (2.12–4.74)). Likewise, those industry workers who were not using personal protective equipment were 2.48 times more likely to have occupational injury compared with those who were using PPE (AOR = 2.48, 95% CI = (1.74–3.56); (Table 4)).

## 4. Discussion

The main aim of this study was to determine the prevalence of occupational injury and its associated factors among industrial workers in the Bahir Dar city. The highest occupational injury was registered in large-scale industries (69.4%) followed by medium-scale (56.5%) and small-scale (50.6%) industries within the last 12 months. The finding suggests that there is a difference in the occurrence of occupational injury between large-, medium-, and small-scale industries. This might be because the larger industries may have different machines and equipment a lot more than medium- and small-scale industries increasing the probability of getting injured for the workers.

The overall prevalence of occupational injury in the Bahir Dar city was 509 (63.4%). This finding is higher than the findings in Ethiopia [17, 19, 22–24]. Though the value for the overall prevalence lays between the prevalence of large-, medium-, and small scale industries, it is skewed to the value for large-scale industries' prevalence. Therefore, looking at the prevalence of occupational injury in each type of industries gives the true picture of the problem, and hence, managers of the industries can solve the problem easily.

Among occupationally injured participants, the majority (56.8%) of them had a hand injury. This finding is consistent with the finding from Ethiopia [23, 24]. In the city, the majority of the industries were labor-intensive and mostly operated by hand, and this may have its role in getting hands frequently injured. Regarding the types of injuries, 218 (42.8%) was an abrasion. This finding is consistent with the finding from Arba Minch, Ethiopia [24], but inconsistent with the finding from Addis Ababa, Ethiopia, where the most frequent type of injury was cutting [22]. The difference might be due to differences in the study settings. However, 190 (37.3%) of the injury was due to machinery. This finding is consistent with the finding from Arba Minch, Ethiopia [24].

Among participants who had the occupational injury, 226 (44.4%) of them were not using personal protective equipment. This finding is lower than the findings from Ethiopia [22–24]. As the result tells us, workers were not regularly supervised by their supervisors, and this may have their role in poor use of PPE. Moreover, industries give more attention to production than workers' safety. Amazingly, those industry workers who were not regularly supervised by health and safety personnel were 2.66 times more likely to have occupational injury compared with their counterparts. This finding is consistent with the findings from Arba Minch [19, 24].

Concerning the timing of the injury, 312 (61.3%) were injured in the afternoon. The possible reason for this finding could be in the afternoon, workers may drink alcohol and chew khat after lunch, which in turn could expose them to occupational injury given that there was no observed association between alcohol drinking, khat chewing, and occupational injury in the model.

Male study participants were 3.66 times more likely to have occupational injury compared with females. This finding is consistent with the findings from Ethiopia [17, 19]. As evidenced by this research, khat chewing is common among males than females, exposing males more frequently to occupational injury than females. Moreover, it may also be due to differences in schooling. In Ethiopia, there is a schooling disparity between males and females, and males are highly privileged to get educated than females, which in turn may lead to higher employment of males in industries biasing the association between sex and occupational injury. Moreover, research evidence indicates that those temporarily employed industry workers were also 7.33 times more likely to have occupational injury compared with those permanently employed. The possible justification for the finding might be related to the fact that in one case, temporarily employed worker may not get training about the work process at the entry to the new job, and in the other case, they might feel worried about the sustainability of their job.

In addition to this those industry workers who had not taken training regarding occupational health and safety at the entry to a new job was 3.18 times more likely to have occupational injury compared with those who had taken training regarding occupational health and safety at the entry to a new job. This finding is consistent with the findings from Gondar [17] and Arba Minch, Ethiopia [24].

Moreover, those industry workers who were not using personal protective equipment were 2.48 times more likely to have occupational injury compared with those who were using PPE. This finding is consistent with the findings from Addis Ababa [22–24].

## 5. Conclusion

The prevalence of occupational injury in this research is found to be very high. It is advisable to provide sustainable training and regular occupational health and safety supervision for industrial workers in the city. Moreover, research has to be conducted to know the reasons for the difference in the prevalence of occupational injury between large-, medium-, and small-scale industries.

### 5.1. Limitations of This Research

The one-year prevalence may be underestimated or overestimated due to recall and social desirability bias although much effort was taken to minimize it.

## Figures and Tables

**Table 1:** Sociodemographic characteristics of industrial workers in the Bahir Dar city, Northwest Ethiopia, 2017 (*n* = 509).

Variable	Frequency	Percent
Sex		
Female	212	26.4
Male	591	73.6
Age of respondents, years		
14–29	544	67.7
30–45	213	26.5
>45	46	5.7
Religion		
Orthodox	749	93.3
Muslim	48	6
Protestant	6	0.7
Marital status		
Married	384	47.8
Single	364	45.3
Divorced	40	5
Widowed	15	1.9
Educational status		
Unable to read and write	8	1
Able to read and write	16	2
Primary (1–8)	102	12.7
Secondary (9–12)	158	19.7
Certificate or diploma	378	47
First degree or above	141	17.6
Type of employment		
Permanent	695	86.6
Temporary	108	13.4
Work experience		
≤7 years	536	66.7
>7 years	267	33.3
Monthly salary		
≤US$ 91	403	50.2
>US$ 91	400	49.8

**Table 2:** Types of personal protective equipment used among industrial workers in the Bahir Dar city, 2017 (*n* = 508).

Type of PPE	Frequency	Percent
Glove		
No	337	66.3
Yes	171	33.7
Ear plug		
No	470	92.5
Yes	38	7.5
Respirators		
No	420	82.7
Yes	88	17.3
Helmet		
No	441	86.8
Yes	67	13.2
Cloth		
No	18	3.5
Yes	490	96.5
Goggle		
No	389	76.6
Yes	119	23.4
Face shield		
No	410	80.7
Yes	98	19.3
Boots		
No	390	76.8
Yes	118	23.2
Other		
No	503	99
Yes	5	1

**Table 3:** Distribution of occupational injury by body parts injured among industrial workers in the Bahir Dar city, 2017 (*n* = 509).

Type of Injury	Frequency	Percent
Hand	189	37.1
Eye	94	18.5
Toe	36	7.1
Leg	63	12.4
Finger	32	6.3
Ear	31	6.1
Back	16	3.1
Head	8	1.6
Knee	8	1.6
Tooth	8	1.6
Other	24	4.7

**Table 4:** Bivariable and multivariable analysis of factors affecting occupational injury among industrial workers in the Bahir Dar city, 2017 (*n* = 803).

Variable	Occupational injury		COR with 95% CI	AOR with 95% CI	*P* value
	No	Yes			
Sex					
Female	117	95	1	1	
Male	177	414	2.88 (2.09–3.98)	3.66 (2.53–5.29)	*P* < 0.001^*∗*^
Marital status of respondents					
Married	163	221	1	1	
Unmarried	131	288	1.62 (1.21–2.17)	1.02 (0.72–1.47)	0.881
Type of employment					
Permanent	287	408	1	1	
Temporary	7	101	10.1 (4.65–22.16)	7.33 (3.31–16.22)	*P* < 0.001^*∗*^
Regular supervision on health & safety					
No	200	413	2.02 (1.45–2.82)	2.66 (1.79–3.96)	*P* < 0.001^*∗*^
Yes	95	96	1	1	
Training at entry to a new job					
No	69	167	1.59 (1.15–2.21)	3.18 (2.14–4.74)	*P* < 0.001^*∗*^
Yes	225	342	1	1	
Experience					
≤7 years	170	366	1	1	
>7 years	124	143	0.53 (0.40–0.72)	0.77 (0.52–1.14)	0.188
Personal protective equipment use					
No	80	215	1.96 (1.43–2.67)	2.48 (1.74–3.56)	*P* < 0.001^*∗*^
Yes	214	294	1	1	

Here, the symbol ^*∗*^indicates that the variable significantly associated with occupational injury at *P* value <0.001.

## Data Availability

The SPSS data used to support the findings of this study are available from the corresponding author upon request.
